# COVID-19 Antiviral Prescription Receipt Among Outpatients Aged ≥65 Years — United States, June 1, 2023–September 30, 2025

**DOI:** 10.15585/mmwr.mm7506a1

**Published:** 2026-02-19

**Authors:** Julia Raykin, Ilia Rochin, Ryan Wiegand, Victoria Soto, Afua Nyame-Mireku, Amy Chung, Josephine Mak, Tegan Boehmer, Pragna Patel

**Affiliations:** ^1^Office of Public Health Data, Surveillance, and Technology, CDC; ^2^National Center for Immunization and Respiratory Diseases, CDC.

**Summary**

**What is already known about this topic?**

COVID-19 antiviral use is low among outpatients aged ≥65 years, a population at high risk for severe disease.

**What is added by this report?**

During June 1, 2023–September 30, 2025, 16%–23% of outpatients aged ≥65 years with COVID-19 received an antiviral prescription during periods of low COVID-19 incidence compared with 37%–38% during higher incidence periods. Adults aged 75–84 years and ≥85 years were more likely to receive an antiviral prescription than were those aged 65–74 years.

**What are the implications for public heath practice?**

COVID-19 vaccination and treatment can prevent severe COVID-19 among older adults. Efforts to improve health care provider and patient knowledge regarding the benefits of COVID-19 vaccination and antivirals, especially for older adults, are needed to reduce the risk for severe illness and death.

## Abstract

Adults aged ≥65 years have the highest rates of COVID-19–related hospitalization. Despite the proven benefit of COVID-19 antivirals in preventing severe outcomes, data suggest that their use is low among older adults. To assess factors associated with receipt of an antiviral prescription among adults aged ≥65 years examined in outpatient settings who received a positive SARS-CoV-2 test result or COVID-19 diagnosis during June 1, 2023–September 30, 2025, multivariate logistic regression analysis of Truveta real-time deidentified electronic health record data was performed. The percentage of COVID-19 outpatients aged ≥65 years who received an antiviral prescription was lower in spring 2024 (21%), fall–winter 2024–25 (23%), spring 2025 (16%), and summer 2025 (19%) than during other seasons (range = 37%–38%). Among those persons who received a prescription, 99% received it within 7 days of a positive SARS-CoV-2 test result or COVID-19 diagnosis, and 80% were prescribed nirmatrelvir/ritonavir. Among adults aged ≥65 years, the odds of receiving an antiviral prescription were higher among those aged 75–84 and ≥85 years (adjusted odds ratio [aOR] = 1.09 and 1.11, respectively), Asian (aOR = 1.42) or Hispanic or Latino persons (aOR = 1.24), and those who had received ≥1 COVID-19 vaccine dose (aOR = 1.73) than among adults in other age, racial, ethnic, and vaccination status groups. Persons with at least one comorbidity and rural residents had lower odds of receiving an antiviral prescription. Persons with COVID-19 had higher odds of receiving a COVID-19 antiviral prescription in summer 2024 (aOR = 1.05) compared with other analytic periods. Odds of prescribing were lower during periods of lower COVID-19 incidence. Antivirals might be underprescribed among adults aged ≥65 years, and prescribing rates vary temporally. Encouraging annual COVID-19 vaccination and increased prescribing of antivirals among adults aged ≥65 years with COVID-19 could reduce the risk for severe illness and hospitalization in this population.

## Introduction

SARS-CoV-2 circulates year-round and typically surges during the summer, fall, and winter, resulting in considerable morbidity and mortality (*1*). During 2020–24, the fall–winter (October–February) COVID-19 surges were consistently more severe than were the summer (June–September) surges; however, the 2024 summer surge resulted in more COVID-19 hospitalizations than did the 2024–25 fall–winter surge (*1*). During recent years, the risk for hospitalization has been highest among adults aged ≥65 years, with risk increasing with age (*1*). Adults aged ≥65 years, persons with multiple comorbidities or immunocompromising conditions, and persons who have not received a seasonal COVID-19 vaccine are at highest risk for severe disease (*2*). Studies suggest that the use of COVID-19 antiviral treatment is low, especially among older adults (*3*,*4*). Real-world effectiveness studies have determined that COVID-19 antiviral treatment with nirmatrelvir/ritonavir in outpatient settings reduces the risk for severe outcomes (*5*). To better understand variations in prescribing of antivirals, this study analyzed data from Truveta, a real-time collection of deidentified electronic health records from more than 30 U.S. health systems. This analysis evaluated the frequency of antiviral prescriptions and the factors associated with their receipt among adults aged ≥65 years with COVID-19 who were examined in outpatient settings during June 2023–September 2025.

## Methods

### Data Source

This retrospective cohort study examined deidentified electronic health records from Truveta, which collects data from health care organizations nationwide, including approximately 120 million patients receiving care at approximately 20,000 clinics and 900 hospitals in the United States. Data are transformed using the Truveta Data Model and standardized through the Truveta Language Model. Data from June 1, 2023, through September 30, 2025, were extracted in January 2026 for this analysis. COVID-19 cases were identified among outpatients aged ≥65 years, using one of two criteria: 1) a laboratory-confirmed molecular or antigen SARS-CoV-2 test result or 2) a health care encounter with an *International Classification of Diseases, Tenth Revision, Clinical Modification* (ICD-10-CM) diagnosis code for COVID-19 (U07.1). The index date was defined as the earliest date of documentation of either of these criteria. The analysis was limited to persons identified as outpatients in primary care settings (by Truveta code 1065216 for ambulatory patients) who had not had an inpatient encounter in the month preceding the index date. Only one encounter with a COVID-19 diagnosis per analysis period was included for any individual patient, although a given patient could be included in multiple analysis periods. 

A COVID-19 antiviral prescription documented up to 7 days after the index date in any outpatient setting was considered an indication of treatment with one of the following: nirmatrelvir/ritonavir, remdesivir, or molnupiravir. COVID-19 treatment initiation was approximated by the date of the prescription. COVID-19 cases that were not diagnosed during June 1, 2023–September 30, 2025, were excluded. Persons aged <65 years, those who had received a positive SARS-CoV-2 test result or diagnosis for COVID-19 during the 6 months preceding the index date, and patients who received treatment but did not have a COVID-19 diagnosis or positive SARS-CoV-2 test result during the 6 months preceding the index date were also excluded, because the dataset does not capture symptomatology consistently enough to assess the receipt of empiric treatment.

### Patient Characteristics and Analysis Periods

Persons with COVID-19 were described by age group, sex, race, ethnicity, COVID-19 vaccination status, comorbidities, use of immunosuppressive medication, and receipt of a COVID-19 antiviral prescription (overall and by medication) for each analysis period. Periods were determined by season and then categorized on the basis of incidence into surge and nonsurge periods of SARS-CoV-2 circulation: summer 2023 (June 1–September 30, 2023), fall–winter 2023–24 (October 1, 2023–February 29, 2024), spring 2024 (March 1–May 31, 2024), summer 2024 (June 1–September 30, 2024), fall–winter 2024–25 (October 1, 2024–February 28, 2025), spring 2025 (March 1–May 31, 2025), and summer 2025 (June 1–September 30, 2025). Persons with documentation of receipt of a COVID-19 vaccine dose within the preceding 6 months were considered to be vaccinated. Receipt of vaccination was ascertained using Current Procedural Terminology (CPT) codes, CVX codes, National Drug Codes, or RxNorm codes. Underlying medical conditions were defined by ICD-10-CM, CPT, or Healthcare Common Procedure Coding System codes documented since 2016. The main outcome was receipt of COVID-19 antiviral prescription within 7 days of the index date.

### Statistical Analysis

Seasonal variation in the proportion of patients receiving an antiviral prescription was analyzed using a generalized linear model with a binomial error distribution and logit link. Adjusted odds ratios (aORs) for receipt of antiviral prescription were estimated using multivariable logistic regression models across all analysis periods after controlling for age, sex, race, ethnicity, comorbidities, COVID-19 vaccination status, urbanicity,* and analytic period; 95% CIs were used to estimate uncertainty. Analyses were conducted using R software (version 4.2.1; R Foundation). This activity was reviewed by CDC, deemed research not involving human subjects, and was conducted consistent with applicable federal law and CDC policy.^†^

## Results

### Patient Characteristics and Receipt of COVID-19 Antiviral Prescriptions

A total of 482,456 patient encounters with a COVID-19 diagnosis were assessed, including 147,715 (31%) during which a COVID-19 antiviral was prescribed and 334,741 (69%) during which one was not. The number of patient encounters in each analysis period ranged from 33,926 (spring 2025) to 117,038 (fall–winter 2023–24) (Table 1).

**TABLE 1:** Characteristics of adults aged ≥65 years with COVID-19 who received COVID-19 antiviral prescriptions in outpatient care settings, by analysis period* — Truveta database, United States, June 2023–September 2025

Characteristic	No. (%)													
	Summer 2023 n = 74,953		Fall–winter 2023–24 n = 117,038		Spring 2024 n = 36,153		Summer 2024 n = 94,132		Fall–winter 2024–25 n = 70,371		Spring 2025 n = 33,926		Summer 2025 n = 55,883	
	Prescribed	Not prescribed	Prescribed	Not prescribed	Prescribed	Not prescribed	Prescribed	Not prescribed	Prescribed	Not prescribed	Prescribed	Not prescribed	Prescribed	Not prescribed
**Total (N = 482,456)**	**27,482 (36.7)**	**47,471 (63.3)**	**43,951 (37.6)**	**73,087 (62.4)**	**7,760 (21.5)**	**28,393 (78.5)**	**35,414 (37.6)**	**58,718 (62.4)**	**16,651 (23.7)**	**53,720 (76.3)**	**5,610 (16.5)**	**28,316 (83.5)**	**10,847 (19.4)**	**45,036 (80.6)**
**Median age, yrs (IQR)**	73 (69–79)	73 (69–79)	74 (69–79)	73 (69–79)	74 (69–79)	73 (69–79)	74 (69–79)	73 (69–79)	74 (69–79)	73 (69–79)	74 (69–80)	73 (69–79)	73 (69–79)	73 (69–79)
**Age group, yrs**														
65–74	15,275 (55.6)	26,976 (56.8)	23,538 (53.6)	40,667 (55.6)	4,130 (53.2)	16,011 (56.4)	18,991 (53.6)	32,181 (54.8)	8,914 (53.5)	30,195 (56.2)	2,836 (50.6)	15,714 (55.5)	5,943 (54.8)	24,948 (55.4)
75–84	9,544 (34.7)	15,791 (33.3)	15,356 (34.9)	24,532 (33.6)	2,788 (35.9)	9,567 (33.7)	12,556 (35.5)	20,434 (34.8)	5,963 (35.8)	18,158 (33.8)	2,075 (37.0)	9,707 (34.3)	3,899 (35.9)	15,633 (34.7)
≥85	2,663 (9.7)	4,704 (9.9)	5,057 (11.5)	7,888 (10.8)	842 (10.9)	2,815 (9.9)	3,867 (10.9)	6,103 (10.4)	1,774 (10.7)	5,367 (10.0)	699 (12.5)	2,895 (10.2)	1,005 (9.3)	4,455 (9.9)
**Sex**														
Female	15,898 (57.85)	27,299 (57.51)	25,465 (57.94)	41,690 (57.04)	4,522 (58.27)	15,794 (55.63)	20,570 (58.08)	33,603 (57.23)	9,861 (59.22)	30,137 (56.10)	3,374 (60.14)	16,120 (56.93)	6,397 (58.97)	25,832 (57.36)
Male	11,583 (42.15)	20,165 (42.48)	18,477 (42.04)	31,394 (42.95)	3,238 (41.73)	12,597 (44.37)	14,841 (41.91)	25,107 (42.76)	6,788 (40.77)	23,581 (43.90)	2,236 (39.86)	12,195 (43.07)	4,445 (40.98)	19,198 (42.63)
Unknown	1 (—)	7 (—)	9 (—)	3 (—)	0 (—)	2 (—)	3 (—)	8 (—)	2 (—)	2 (—)	0 (—)	1 (—)	5 (—)	6 (—)
**Race and ethnicity**														
AI/AN	14 (—)	26 (—)	13 (—)	30 (—)	6 (—)	17 (—)	18 (—)	32 (—)	4 (—)	14 (—)	1 (—)	15 (—)	1 (—)	17 (—)
Asian	1,245 (4.53)	1,349 (2.84)	1,591 (3.62)	1,660 (2.27)	343 (4.42)	782 (2.75)	1,354 (3.82)	1,487 (2.53)	369 (2.22)	1,171 (2.18)	155 (2.76)	721 (2.55)	348 (3.21)	1,355 (3.01)
Black or African American, NH	1,463 (5.32)	3,400 (7.16)	2,310 (5.26)	4,642 (6.35)	412 (5.31)	1,617 (5.70)	2,192 (6.19)	4,072 (6.93)	1,026 (6.16)	3,232 (6.02)	472 (8.41)	1,701 (6.01)	978 (9.02)	2,554 (5.67)
Hispanic or Latino	1,180 (4.29)	1,867 (3.93)	1,801 (4.10)	2,505 (3.43)	287 (3.70)	969 (3.41)	1,719 (4.85)	2,249 (3.83)	922 (5.54)	2,435 (4.53)	254 (4.53)	1,043 (3.68)	710 (6.55)	2,547 (5.66)
NH/PI	45 (0.16)	53 (0.11)	36 (—)	57 (—)	14 (0.18)	34 (0.12)	48 (0.14)	78 (0.13)	4 (—)	55 (0.10)	0 (—)	29 (0.10)	20 (0.18)	63 (0.14)
White, NH	20,316 (73.92)	35,653 (75.10)	33,313 (75.8)	56,479 (77.28)	5,811 (74.88)	22,082 (77.77)	26,078 (73.64)	44,324 (75.49)	12,529 (75.24)	41,046 (76.41)	4,085 (72.82)	21,825 (77.08)	7,463 (68.80)	33,000 (73.27)
Other	521 (1.90)	733 (1.54)	827 (1.88)	1,121 (1.53)	148 (1.91)	406 (1.43)	655 (1.85)	869 (1.48)	114 (0.68)	395 (0.74)	83 (1.48)	438 (1.55)	85 (0.78)	399 (0.89)
Unknown	2,698 (9.82)	4,390 (9.25)	4,060 (9.24)	6,593 (9.02)	739 (9.52)	2,486 (8.76)	3,350 (9.46)	5,607 (9.55)	1,683 (10.11)	5,372 (10.00)	560 (9.98)	2,544 (8.98)	1,242 (11.45)	5,101 (11.33)
**Chronic medical condition**														
Cancer	6,242 (22.71)	11,151 (23.49)	10,089 (22.96)	17,430 (23.85)	1,934 (24.92)	7,213 (25.40)	8,492 (23.98)	14,433 (24.58)	3,886 (23.34)	13,557 (25.24)	1,399 (24.94)	7,303 (25.79)	2,528 (23.31)	11,255 (24.99)
Cardiac disease	14,666 (53.37)	28,040 (59.07)	24,851 (56.54)	44,323 (60.64)	4,524 (58.30)	17,898 (63.04)	20,491 (57.86)	35,655 (60.72)	9,737 (58.48)	33,884 (63.08)	3,381 (60.27)	18,283 (64.57)	6,310 (58.17)	27,924 (62.00)
Cerebrovascular	4,453 (16.20)	8,496 (17.90)	7,958 (18.11)	13,757 (18.82)	1,441 (18.57)	5,432 (19.13)	6,386 (18.03)	11,187 (19.05)	3,015 (18.11)	10,370 (19.30)	1,057 (18.84)	5,604 (19.79)	1,955 (18.02)	8,646 (19.20)
Chronic liver disease	2,914 (10.60)	5,026 (10.59)	4,782 (10.88)	7,941 (10.87)	910 (11.73)	3,285 (11.57)	4,265 (12.04)	6,830 (11.63)	1,994 (11.98)	6,346 (11.81)	679 (12.10)	3,600 (12.71)	1,397 (12.88)	5,785 (12.85)
Chronic pulmonary disease	9,231 (33.59)	16,523 (34.81)	16,197 (36.85)	27,137 (37.13)	2,969 (38.26)	10,646 (37.50)	13,323 (37.62)	21,696 (36.95)	6,594 (39.60)	20,474 (38.11)	2,324 (41.43)	11,066 (39.08)	4,206 (38.78)	17,459 (38.77)
Diabetes mellitus	7,385 (26.87)	13,995 (29.48)	12,466 (28.36)	21,517 (29.44)	2,126 (27.40)	8,453 (29.77)	10,265 (28.99)	17,216 (29.32)	4,995 (30.00)	16,007 (29.80)	1,745 (31.11)	8,635 (30.50)	3,297 (30.40)	13,473 (29.92)
Disability	8,205 (29.86)	13,272 (27.96)	13,762 (31.31)	21,519 (29.44)	2,630 (33.89)	8,657 (30.49)	11,691 (33.01)	17,779 (30.28)	5,522 (33.16)	16,829 (31.33)	1,905 (33.96)	9,323 (32.92)	3,492 (32.19)	15,167 (33.68)
HIV	35 (0.13)	107 (0.23)	60 (0.14)	130 (0.18)	9 (0.12)	58 (0.20)	59 (0.17)	104 (0.18)	33 (0.20)	121 (0.23)	14 (0.25)	75 (0.26)	17 (0.16)	105 (0.23)
Mental health disorder	7,199 (26.20)	11,447 (24.11)	12,052 (27.42)	18,611 (25.46)	2,131 (27.46)	7,189 (25.32)	9,855 (27.83)	15,152 (25.80)	4,756 (28.56)	13,871 (25.82)	1,593 (28.40)	7,692 (27.16)	2,876 (26.51)	12,210 (27.11)
Neurologic disease/dementia	5,668 (20.62)	9,501 (20.01)	10,003 (22.76)	15,129 (20.70)	1,826 (23.53)	5,609 (19.75)	8,150 (23.01)	12,226 (20.82)	3,780 (22.70)	11,218 (20.88)	1,402 (24.99)	6,381 (22.53)	2,237 (20.62)	10,072 (22.36)
Obesity	5,958 (21.68)	10,389 (21.88)	10,327 (23.50)	16,940 (23.18)	1,766 (22.76)	6,910 (24.34)	8,478 (23.94)	13,845 (23.58)	4,340 (26.06)	13,780 (25.65)	1,460 (26.02)	7,480 (26.42)	2,853 (26.30)	11,526 (25.59)
Physical inactivity	458 (1.67)	625 (1.32)	807 (1.84)	1,196 (1.64)	151 (1.95)	419 (1.48)	855 (2.41)	1,076 (1.83)	423 (2.54)	1,100 (2.05)	150 (2.67)	575 (2.03)	392 (3.61)	1,037 (2.30)
Renal disease	6,465 (23.52)	11,941 (25.15)	11,107 (25.27)	18,974 (25.96)	1,952 (25.15)	7,550 (26.59)	9,178 (25.92)	15,230 (25.94)	4,533 (27.22)	14,327 (26.67)	1,581 (28.18)	7,711 (27.23)	2,944 (27.14)	11,951 (26.54)
Smoking	6,416 (23.35)	11,766 (24.79)	10,948 (24.91)	19,201 (26.27)	1,966 (25.34)	7,551 (26.59)	8,555 (24.16)	14,929 (25.42)	4,100 (24.62)	14,690 (27.35)	1,367 (24.37)	8,034 (28.37)	2,293 (21.14)	11,996 (26.64)
Steroid medications	1,267 (4.61)	2,069 (4.36)	2,154 (4.90)	3,465 (4.74)	411 (5.30)	1,310 (4.61)	1,768 (4.99)	2,715 (4.62)	818 (4.91)	2,666 (4.96)	294 (5.24)	1,489 (5.26)	476 (4.39)	2,305 (5.12)
Other immune-modulating medication	259 (0.94)	590 (1.24)	440 (1.00)	885 (1.21)	71 (0.91)	401 (1.41)	329 (0.93)	710 (1.21)	183 (1.10)	690 (1.28)	61 (1.09)	397 (1.40)	97 (0.89)	586 (1.30)
Transplant recipient	185 (0.67)	410 (0.86)	322 (0.73)	690 (0.94)	58 (0.75)	290 (1.02)	251 (0.71)	537 (0.91)	150 (0.90)	502 (0.93)	49 (0.87)	291 (1.03)	89 (0.82)	422 (0.94)
Tuberculosis	137 (0.50)	171 (0.36)	156 (0.35)	239 (0.33)	36 (0.46)	105 (0.37)	164 (0.46)	221 (0.38)	63 (0.38)	193 (0.36)	21 (0.37)	106 (0.37)	36 (0.33)	184 (0.41)
**No. of comorbidities^†^**														
None	3,780 (13.75)	5,006 (10.55)	5,296 (12.05)	7,086 (9.70)	900 (11.60)	2,115 (7.45)	3,879 (10.95)	5,833 (9.93)	1,811 (10.88)	4,384 (8.16)	588 (10.48)	2,005 (7.08)	1,333 (12.29)	4,075 (9.05)
1	4,083 (14.86)	6,978 (14.70)	5,720 (13.01)	10,079 (13.79)	981 (12.64)	3,897 (13.73)	4,548 (12.84)	7,909 (13.47)	2,114 (12.70)	6,875 (12.80)	675 (12.03)	3,415 (12.06)	1,348 (12.43)	5,567 (12.36)
2	4,244 (15.44)	8,056 (16.97)	6,726 (15.30)	12,114 (16.57)	1,181 (15.22)	4,870 (17.15)	5,410 (15.28)	9,481 (16.15)	2,471 (14.84)	8,727 (16.25)	778 (13.87)	4,594 (16.22)	1,566 (14.44)	6,810 (15.12)
≥3	15,375 (55.95)	27,431 (57.78)	26,209 (59.63)	43,808 (59.94)	4,698 (60.54)	17,511 (61.67)	21,577 (60.93)	35,495 (60.45)	10,255 (61.59)	33,734 (62.80)	3,569 (63.62)	18,302 (64.63)	6,600 (60.85)	28,584 (63.47)
**No. of COVID-19 vaccine doses received in last 6 months**														
None recorded	24,946 (90.77)	45,417 (95.67)	34,206 (77.83)	63,631 (87.06)	5,975 (77.00)	25,013 (88.10)	32,486 (91.73)	56,252 (95.80)	13,558 (81.42)	45,933 (85.50)	5,015 (89.39)	26,072 (92.08)	10,553 (97.29)	42,516 (94.40)
1	2,530 (9.21)	2,050 (4.32)	9,389 (21.36)	9,168 (12.54)	1,768 (22.78)	3,340 (11.76)	2,880 (8.13)	2,435 (4.15)	3,023 (18.16)	7,628 (14.20)	592 (10.55)	2,238 (7.90)	289 (2.66)	2,438 (5.41)
2	6 (—)	4 (—)	356 (0.81)	288 (0.39)	17 (0.22)	40 (0.14)	48 (0.14)	31 (—)	70 (0.42)	159 (0.30)	3 (—)	6 (—)	5 (—)	82 (0.18)
**Rural/Urban classification^§^**														
Rural	1,154 (4.20)	2,772 (5.84)	2,277 (5.18)	4,440 (6.07)	283 (3.65)	1,727 (6.08)	1,563 (4.41)	3,288 (5.60)	988 (5.93)	3,321 (6.18)	243 (4.33)	1,552 (5.48)	405 (3.73)	2,887 (6.41)
Urban	25,641 (93.30)	43,047 (90.68)	40,304 (91.70)	65,799 (90.03)	7,266 (93.63)	25,704 (90.53)	32,939 (93.01)	53,420 (90.98)	15,233 (91.48)	48,888 (91.01)	5,223 (93.10)	25,865 (91.34)	10,221 (94.23)	41,076 (91.21)
Unknown	687 (2.50)	1,652 (3.48)	1,370 (3.12)	2,848 (3.90)	211 (2.72)	962 (3.39)	912 (2.58)	2,010 (3.42)	430 (2.58)	1,511 (2.81)	144 (2.57)	899 (3.17)	221 (2.04)	1,073 (2.38)
**HHS region^¶^**														
1	36 (0.13)	43 (—)	57 (0.13)	102 (0.14)	3 (—)	35 (0.12)	29 (—)	62 (0.11)	27 (0.16)	62 (0.12)	9 (0.16)	20 (—)	10 (—)	34 (—)
2	761 (2.77)	4,614 (9.72)	1,714 (3.90)	7,875 (10.77)	313 (4.03)	2,625 (9.25)	995 (2.81)	4,904 (8.35)	681 (4.09)	5,081 (9.46)	394 (7.02)	2,897 (10.23)	450 (4.15)	3,292 (7.31)
3	523 (1.90)	3,005 (6.33)	1,112 (2.53)	3,897 (5.33)	179 (2.31)	1,616 (5.69)	767 (2.17)	2,823 (4.81)	929 (5.58)	5,578 (10.38)	204 (3.64)	2,198 (7.76)	524 (4.83)	3,948 (8.77)
4	5,963 (21.70)	8,485 (17.87)	9,200 (20.93)	11,400 (15.60)	1,548 (19.95)	4,010 (14.12)	8,222 (23.22)	10,920 (18.60)	3,451 (20.73)	8,337 (15.52)	1,440 (25.67)	4,174 (14.74)	3,788 (34.92)	7,828 (17.38)
5	1,473 (5.36)	6,490 (13.67)	4,369 (9.94)	11,936 (16.33)	691 (8.90)	5,462 (19.24)	2,787 (7.87)	8,847 (15.07)	2,781 (16.70)	10,350 (19.27)	619 (11.03)	4,937 (17.44)	1,080 (9.96)	6,466 (14.36)
6	3,965 (14.43)	8,358 (17.61)	7,453 (16.96)	12,806 (17.52)	1,116 (14.38)	4,576 (16.12)	5,350 (15.11)	10,344 (17.62)	3,249 (19.51)	7,612 (14.17)	1,378 (24.56)	3,577 (12.63)	3,007 (27.72)	4,045 (8.98)
7	326 (1.19)	1,775 (3.74)	768 (1.75)	2,643 (3.62)	128 (1.65)	1,110 (3.91)	601 (1.70)	2,020 (3.44)	457 (2.74)	2,041 (3.80)	101 (1.80)	1,220 (4.31)	195 (1.80)	1,523 (3.38)
8	404 (1.47)	330 (0.70)	616 (1.40)	882 (1.21)	147 (1.89)	850 (2.99)	682 (1.93)	1,274 (2.17)	322 (1.93)	1,231 (2.29)	75 (1.34)	738 (2.61)	115 (1.06)	1,155 (2.56)
9	5,437 (19.78)	5,648 (11.90)	7,469 (16.99)	8,273 (11.32)	1,314 (16.93)	3,046 (10.73)	6,696 (18.91)	6,797 (11.58)	2,284 (13.72)	5,360 (9.98)	655 (11.68)	3,504 (12.37)	926 (8.54)	7,236 (16.07)
10	7,775 (28.29)	6,620 (13.95)	9,484 (21.58)	9,560 (13.08)	2,100 (27.06)	3,853 (13.57)	8,165 (23.06)	8,087 (13.77)	1,849 (11.10)	5,645 (10.51)	559 (9.96)	3,838 (13.55)	390 (3.60)	7,657 (17.00)
Unknown	819 (2.98)	2,103 (4.43)	1,709 (3.89)	3,713 (5.08)	221 (2.85)	1,210 (4.26)	1,120 (3.16)	2,640 (4.50)	621 (3.73)	2,423 (4.51)	176 (3.14)	1,213 (4.28)	362 (3.34)	1,852 (4.11)

**Abbreviations:** AI/AN = American Indian or Alaska Native; HHS = U.S. Department of Health and Human Services; NH = non-Hispanic; NH/PI = Native Hawaiian or Pacific Islander.

* Summer 2023 = June 1–September 30, 2023; fall–winter 2023–2024 = October 1, 2023–February 29, 2024; spring 2024 = March 1–May 31, 2024; summer 2024 = June 1–September 30, 2024; fall–winter 2024–2025 = October 1, 2024–February 28, 2025; spring 2025 = March 1–May 31, 2025; summer 2025 = June 1–September 30, 2025.

**^†^** For chronic medical conditions (nonexclusive categories), each condition was compared in a 2x2 table using total number of patients with this comorbidity and total number of patients without this comorbidity by treatment status.

**^§^** Rural/urban classification is defined by residential address based on Federal Office of Rural Health Policy Data files from 2024, which contain all zip codes classified as rural areas.

^¶^ HHS regional offices: 1 = Boston, Massachusetts; 2 = New York, New York; 3 = Philadelphia, Pennsylvania; 4 = Atlanta, Georgia; 5 = Chicago, Illinois; 6 = Dallas, Texas; 7 = Kansas City, Missouri; 8 = Denver, Colorado; 9 = San Francisco, California; and 10 = Seattle, Washington.

The percentage of COVID-19 patients who received an antiviral prescription varied across analysis periods: the percentage who received an antiviral prescription during fall–winter 2024–25 (23.4%) was higher than that during spring 2024 (21.5%), spring 2025 (16.5%), and summer 2025 (19.4%), and lower than that during the summer 2023 (36.7%), fall–winter 2023–24 (37.6%), and summer 2024 (37.6%) analysis periods (Figure). Among COVID-19 outpatients aged ≥65 years who received a COVID-19 antiviral prescription, 99% received it within 7 days of a positive SARS-CoV-2 test result or COVID-19 diagnosis; 80% of treated patients received nirmatrelvir/ritonavir, 13% received molnupiravir, and 7% received remdesivir across seasons (Supplementary Figure).

**FIGURE:**
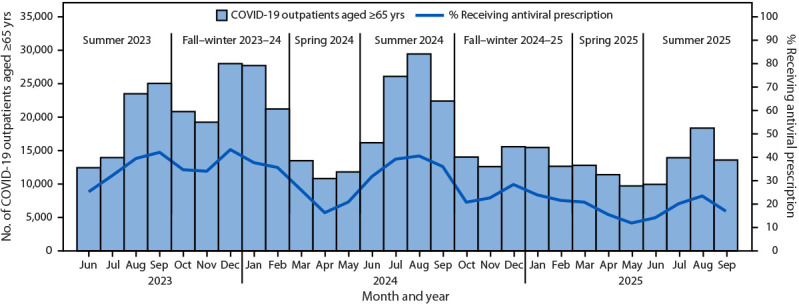
Percentage of patients aged ≥65 years with COVID-19* who received a COVID-19 antiviral prescription in an outpatient setting, by analysis period^†^ — Truveta database,^§^ United States, June 1, 2023–September 30, 2025 * Receipt of a positive SARS-CoV-2 molecular or antigen test result or a health care encounter with an *International Classification of Diseases, Tenth Revision, Clinical Modification* COVID-19 diagnosis code. ^†^ Summer 2023 = June 1–September 30, 2023; fall–winter 2023–24 = October 1, 2023–February 29, 2024; spring 2024 = March 1–May 31, 2024; summer 2024 = June 1–September 30, 2024; fall–winter 2024–25 = October 1, 2024–February 28, 2025; spring 2025 = March 1–May 31, 2025; summer 2025 = June 1–September 30, 2025. ^§^ EHR data and analytics | Truveta

### Factors Associated with COVID-19 Antiviral Prescriptions

For each period examined, significant differences in age, race, ethnicity, number of COVID-19 vaccine doses received, number of comorbidities, and urbanicity were observed between COVID-19 outpatients aged ≥65 years who did and did not receive a COVID-19 antiviral prescription in descriptive analysis (Table 1). In multivariable analysis, compared with adults aged 65–74 years, the odds of receiving an antiviral prescription were higher among adults aged 75–84 years and ≥85 years (aOR = 1.09 and 1.11, respectively) (Table 2). Compared with non-Hispanic White persons, the odds of receiving an antiviral prescription were higher among Asian persons (aOR = 1.42) and Hispanic or Latino persons (aOR = 1.24). Compared with persons who did not receive a COVID-19 vaccine dose during the preceding 6 months, the odds of receiving an antiviral prescription among those who had received 1 or 2 doses were higher (aOR = 1.73). The odds of receiving a COVID-19 antiviral prescription were lower among persons with one comorbidity (aOR = 0.76) and at least two comorbidities (aOR = 0.75) than among those with no comorbidities. Compared with persons who live in urban settings, the odds of receiving an antiviral prescription were lower among those who lived in rural communities (aOR = 0.81). Among persons with COVID-19, the odds of receiving an antiviral prescription were higher in summer 2024 (aOR = 1.05) and lower in spring 2024 (aOR = 0.45), fall–winter 2024–25 (aOR = 0.51), spring 2025 (aOR = 0.34), and summer 2025 (aOR = 0.41) compared with summer 2023.

**TABLE 2:** Factors associated with receipt of antiviral prescriptions among adults aged ≥65 with COVID-19* in outpatient care settings — Truveta database, United States, June 2023–September 2025

Characteristic	Adjusted odds ratio (95% CI)^†^
**Age group, yrs**	
65–74	Referent
75–84	1.09 (1.07–1.10)
≥85	1.11 (1.09–1.14)
**Sex**	
Female	Referent
Male	0.95 (0.94–0.96)
**Race and ethnicity**	
White, non-Hispanic	Referent
American Indian/Alaska Native	0.85 (0.62–1.18)
Asian	1.42 (1.37–1.47)
Black or African American, non-Hispanic	0.97 (0.95–1.00)
Hispanic or Latino	1.24 (1.20–1.28)
Native Hawaiian or Pacific Islander	1.12 (0.93–1.35)
Other	1.21 (1.15–1.28)
Unknown	1.03 (1.01–1.05)
**No. of comorbidities**	
0	Referent
1	0.76 (0.74–0.78)
≥2	0.75 (0.74–0.77)
**No. of COVID-19 vaccine doses in last 6 months**	
0	Referent
1 or 2	1.73 (1.70–1.77)
**Rural/Urban classification^§^**	
Urban	Referent
Rural	0.81 (0.79–0.84)
**Analysis period^¶^**	
Summer 2023	Referent
Fall–winter 2023–24	0.98 (0.96–1.00)
Spring 2024	0.45 (0.44–0.46)
Summer 2024	1.05 (1.03–1.07)
Fall–winter 2024–25	0.51 (0.49–0.52)
Spring 2025	0.34 (0.33–0.35)
Summer 2025	0.41 (0.40–0.43)

* Receipt of a positive SARS-CoV-2 molecular or antigen test result or a health care encounter with an *International Classification of Diseases, Tenth Revision, Clinical Modification* COVID-19 diagnosis code.

**^†^** The logistic regression models were estimated using a generalized linear model with a binomial family and a logit link. All models included the variables reported as main effects. For all categorical covariates, the level treated as the referent category is listed in the table. Adjusted odds ratios are interpreted relative to this referent.

^§^ Rural/urban classification is defined by residential address based on Federal Office of Rural Health Policy Data files from 2024, which contain all zip codes classified as rural areas.

^¶^ Summer 2023 = June 1–September 30, 2023; fall–winter 2023–2024 = October 1, 2023–February 29, 2024; spring 2024 = March 1–May 31, 2024; summer 2024 = June 1–September 30, 2024; fall–winter 2024–2025 = October 1, 2024–February 28, 2025; spring 2025 = March 1–May 31, 2025; summer 2025 = June 1–September 30, 2025.

## Discussion

This study of a large national database of COVID-19 cases suggests that antivirals are underprescribed to older adults, a population at increased risk for severe disease (*1*). These findings are consistent with those described in a 2023 analysis of receipt of antiviral medication among outpatients aged ≥65 years with COVID-19. However, unlike the 2023 study, this analysis found that the percentage who received an antiviral increased with increasing age (*3*).

Increased receipt of an antiviral prescription with increasing age suggests an improvement in health care provider recommendations for antivirals among older adults with COVID-19, particularly because the risk for severe disease increases with increasing age (*1*). In this analysis, persons with comorbidities were less likely to receive a COVID-19 antiviral prescription than were those without comorbidities. This might reflect complicated medication profiles of older adults as well as health care provider concerns about the potential for drug interactions. However, because persons with comorbidities are at increased risk for severe disease, considering treatment for these persons can help prevent morbidity and mortality. Increased receipt of COVID-19 antiviral prescriptions among persons who had received the COVID-19 vaccine suggests that persons who engage in preventive care might be more likely to seek treatment when they are ill. COVID-19 vaccination rates are lower now than in previous years (*6*). Therefore, recommending primary prevention strategies to protect against severe COVID-19 to older adults, including COVID-19 vaccination, can reduce hospitalizations and deaths. For additional protection, consideration of early outpatient treatment for older adults who develop mild to moderate COVID-19 can help prevent disease progression.

The receipt of an antiviral prescription varied across periods and seemed to correspond with intensity of SARS-CoV-2 circulation, suggesting that perception of risk related to SARS-CoV-2 activity rather than individual risk might be a factor in clinical decision-making about treatment (*7*). Health care providers and patients should be educated about the benefits and risks of COVID-19 antiviral treatment. Accurate assessment of risk for severe disease and options for treatment are important for early initiation to prevent severe outcomes. Barriers to COVID-19 treatment, particularly nirmatrelvir/ritonavir, include concerns about drug interactions (*8*). However, studies suggest these interactions can be safely managed (*9*). Improved awareness of COVID-19 antiviral patient access programs could improve availability.

### Limitations

The findings in this report are subject to at least five limitations. First, these data are not representative of the general U.S. population because they reflect only the health care–seeking population. Second, selection bias might have resulted from exclusion of persons who did not have electronic health record documentation of a positive SARS-CoV-2 test result or COVID-19 diagnosis and likely resulted in underascertainment of COVID-19 cases. Third, lack of symptom data affected the accurate assessment of eligibility for treatment. Fourth, data are limited to those that were documented by the clinician, likely resulting in incomplete data on some variables such as vaccination status. Finally, contraindications to treatment, which might have biased the estimate of eligibility for treatment, could not be examined.

### Implications for Public Health Practice

This study suggests that COVID-19 antivirals are likely underprescribed to outpatients aged ≥65 years with COVID-19 and that their use varies with disease transmission intensity. Increased perception of individual risk for severe COVID-19, which is largely driven by age, might increase antiviral prescribing and prevent significant morbidity and mortality among older adults (*10*). COVID-19 vaccination and treatment remain essential interventions for preventing severe COVID-19 among older adults throughout the year, because individual risk for progression to severe disease remains even when SARS-CoV-2 circulation is low. Public health efforts to improve health care provider and patient knowledge of the benefits of COVID-19 vaccination and antivirals, especially for outpatients aged ≥65 years with COVID-19, are needed to prevent severe outcomes.

## Footnotes

* Because geographic variables were not included in the dataset, whether patients lived in an urban or rural area was determined by whether they lived within a city in their U.S. Department of Health and Human Services region. Rural/urban classification is defined by residential address based on Federal Office of Rural Health Policy Data files from 2024, which contain all zip codes classified as rural areas.

† 45 C.F.R. part 46; 21 C.F.R. part 56; 42 U.S.C. Sect. 241(d), 5 U.S.C. Sect. 552a, 44 U.S.C. Sect. 3501 et seq.
